# Optimizing Antibiotic Choice, Administration, and Duration in NSTI Treatment

**DOI:** 10.3390/bioengineering12070691

**Published:** 2025-06-24

**Authors:** Devorah Howell, Rachael Edgin, Aliya Rehman, Ronald Rabinowitz

**Affiliations:** 1 University of Maryland Medical Center, Division of Infectious Diseases, R. Adams Cowley Shock Trauma Center, Baltimore, MD 21201, USA; redgin@som.umaryland.edu; 2 University of Maryland School of Medicine, Department of Medicine, Division of Infectious Diseases, R. Adams Cowley Shock Trauma Center, Baltimore, MD 21201, USA; aliya.rehman@som.umaryland.edu (A.R.); rrabinowitz@som.umaryland.edu (R.R.)

**Keywords:** necrotizing soft tissue infections, toxic shock syndrome, extended infusion beta lactam dosing

## Abstract

Necrotizing soft tissue infections (NSTIs) are serious and aggressive infections which can result in significant morbidity and mortality. Both prompt surgical intervention and early antibiotics can decrease patient mortality. Based on microbiology, NSTIs can be categorized into four different types. Type I is polymicrobial, caused by a mix of both anaerobic and aerobic bacteria. Type II is monomicrobial, usually caused by either Streptococcus or Staphylococcus. Type III infections are caused by Gram-negative bacteria, often marine-related organisms, such as Vibrio. Lastly, Type IV infections are caused by fungi, and they are often associated with trauma. Despite the possibility of all these different pathogens in NSTI, early therapy often consists of a broad Gram-positive antimicrobial such as linezolid or vancomycin, and a broad Gram-negative agent such as piperacillin/tazobactam. Multiple factors including patient comorbidities, environmental exposures, and clinical presentation must also be considered when choosing antimicrobial agents and dosing. Adjunct medical therapies such as intravenous immunoglobulin (IVIG) and the antibiotics clindamycin and linezolid that are aimed at toxin suppression may be utilized to improve outcomes. Microbiological data are critical for optimizing the antimicrobial regimen.

## 1. Introduction

Necrotizing soft tissue infections (NSTIs) are serious and aggressive infections which can result in significant morbidity and mortality. NSTIs can involve the epidermis, dermis, subcutaneous tissue, fascia, and muscle [1]. They usually develop from a break in the skin barrier, which could be related to trauma or surgery [2]. The pathogen rapidly spreads, releasing toxins which activate cytokine release. This leads to micro-vascular thrombosis, causing ischemia and necrosis [3]. Based on microbiology, they can be categorized into four different types. Type I is polymicrobial, caused by a mix of both anaerobic and aerobic bacteria. Type II is monomicrobial, usually caused by either Streptococcus or Staphylococcus. Type III infections are caused by Gram-negative bacteria, often marine-related organisms such as Vibrio. Lastly, Type IV infections are caused by fungi, and they are often associated with trauma [3]. It is critical to quickly differentiate between a more routine skin and soft tissue infection (SSTI) and an NSTI because antimicrobial treatment cannot effectively penetrate dead and dying tissue; thus, early surgical intervention is required [3]. Initially, antimicrobial treatment covers a broad range of the most likely pathogens given the clinical scenario and is later optimized based on microbiological data obtained during surgery. This optimization is crucial to minimize the risk of toxicity, narrow the spectrum of coverage, and ensure that the organisms present are indeed susceptible to the treatment. Sometimes, hyperbaric oxygen (HBO) may also be used as adjunctive therapy. Overall, NSTIs present a challenge in both diagnosis and treatment [4]. This review will delve into the more common types of NSTI and focus on the nuances and newer data for optimizing antimicrobial and adjuvant therapies used to treat this illness.

## 2. Review

### 2.1. Type I

Type I necrotizing soft tissue infections are polymicrobial in nature, involving both aerobic and anaerobic organisms [1]. Among these, the most frequently isolated organisms are Strep and Staph species along with various Enterobacteriaceae and anaerobes [5,6] (Table 1). There is proposed synergistic action between aerobic and anaerobic bacteria with cultures often having between two and six isolates [5]. While this synergism is not completely understood, some hypotheses suggest a mutual protection from phagocytosis, as well as aerobes, which offer initial tissue destruction, providing ample opportunity for anaerobe growth [5,6].

While Type II NSTIs often have no patient predilection, Type I infections are typically seen in patients with underlying risk factors such as advanced age, obesity, diabetes, and immunosuppression [6]. Use of the diabetic medication class known as sodium–glucose cotransporter 2 has also been associated with an increased risk of developing a Type I NSTI, likely due to the enhanced secretion of glucose through urine [7]. Clinical findings manifest similarly to other NSTIs, but Type I infections typically present in the genitalia, perineum, and buttocks, with those affecting genitalia classically referred to as Fournier’s gangrene [6]. Infection tends to originate in the perineum as an abscess and genitourinary tract as a urinary tract infection. These polymicrobial infections may also arise from chronic wounds, such as diabetic foot ulcers or sacral decubiti. Like other NSTI types, the patient often presents with severe pain, out of proportion to initial exam findings, and induration of the skin described as having a “wood-like” texture. A physical exam can reveal erythema, which can occasionally progress to bullae, dark discoloration, and, in the final stages, necrosis. Systemic manifestations can vary but typically include fever, altered mental status, tachycardia, and hypotension [1]. Both prompt surgical intervention, and early antibiotic use can decrease patient mortality.

Several challenges must be considered when choosing initial empiric antibiotics for NSTI such as patient comorbidities, allergies, history of antimicrobial resistance, and overall clinical severity. Because Type I infections are polymicrobial, it is important to use antibiotics with broad coverage while awaiting definitive culture data. Ideally, samples are obtained by the surgeon at the interface of healthy and necrotizing tissues [8]. These are plated on both aerobic and anaerobic culture media. Bacteremia may be present in a subset of patients. Initial combination therapy should include coverage of Gram-positive organisms such as MRSA, Gram-negative organisms, and anaerobes (Table 1). Unless yeast is identified on initial stains or cultures, empiric antifungal therapy is not recommended, as it is seldom a primary pathogen in Type I infections [3]. Emerging resistance in microbes has forced broadened empiric recommendations. For example, in our institution, only about 50% of *Escherichia coli* isolates remain sensitive to ampicillin/sulbactam. The combination of vancomycin and piperacillin/tazobactam has therefore become a widely accepted standard empiric therapy in patients without known extended spectrum beta lactamase resistance (ESBL) [1,2]. Vancomycin offers Gram-positive coverage to include MRSA, and piperacillin/tazobactam provides broad Gram-negative and anaerobe coverage.

While vancomycin and piperacillin/tazobactam can each rarely cause nephrotoxicity, much debate exists in the literature over the possibility of increased risk with the combination [9]. This is further complicated in Type I NSTI patients who are obese as well as critically ill on admission. Multiple studies have shown a higher risk of acute kidney injury (AKI) in obese patients who receive vancomycin [9,10]. In 2020, the Infectious Diseases Society of America (IDSA), along with several pharmacist groups, released updated guidance recommending a change from trough-based vancomycin dosing to dosing by calculating the area under the curve over 24 h to the minimum inhibitory concentration (AUC/MIC). This was shown to help improve the incidence of AKI in morbidly obese patients [11]. For NSTI patients, contrasted scans, multiple operative trips with additional sedation, or anesthetic plus vasopressors likely contribute further to the overall AKI risk. Although a recent large multi-center retrospective study suggested lower rates of AKI with vancomycin in combination with other beta lactams such as cefepime or meropenem, reserving these antibiotics that offer better coverage of resistant Gram-negative pathogens is a priority of antibiotic stewardship efforts worldwide [11]. Substituting vancomycin with a less nephrotoxic alternative thus has become an area of scientific interest.

Our institution sees a high volume of NSTI patients from across the region. Before the adoption of AUC/MIC dosing for vancomycin, based on our internal data, a higher incidence of AKIs was seen in obese patients exposed to this agent. We therefore changed our empiric recommendations to include linezolid instead of vancomycin for patients with a BMI > 35, and one additional risk factor such as diabetes, a recent CT scan with IV contrast, additional nephrotoxic medication, or AKI at time of admission. Linezolid is a newer drug that offers broad Gram-positive coverage to include MRSA and VRE and has lipophilic properties, allowing penetration into soft tissues and suppression of toxins [12]. Linezolid is standardly dosed at 600 mg every 12 h, but both obesity and critical illness can affect the volume of distribution and drug clearance [13]. Several recent studies set out to better understand the pharmacokinetics of linezolid as well as to provide data for optimal dose recommendations in subpopulations of obese and critically ill patients to achieve adequate concentrations in both plasma and targeted tissues [12,13,14]. One of these studies was published from our institution with collected data following the empiric recommendation change from vancomycin to linezolid [12]. Standard linezolid dosing did not maintain sufficiently high antibiotic concentrations to achieve necessary MICs over time for confidence in controlling infection in critically ill obese patients. Weight-based dosing has therefore been proposed for this patient population with dosing every 8 h or every 6 h depending on patient size and the MIC of the pathogen [12].

Similarly to linezolid, beta lactams exhibit time-dependent bacterial killing, which means their effectiveness depends upon the time free plasma drug concentrations remain above the MIC of the pathogen [8]. Two characteristics of severe NSTI alter these concentrations, potentially contributing to negative outcomes. First, local tissue necrosis destroys micro-vessels, impairing drug delivery at the site of infection. Second, more than 50% of NSTI patients present in shock [8]. Septic shock causes vasodilation and endothelial damage, resulting in profound capillary leak into interstitial tissues. In response, medical providers often give large volumes of fluid for resuscitation and use medications that cause vasoconstriction to maintain blood pressure. This significantly alters the volume of distribution and renal clearance. Recent studies have shown that extending the infusion time of beta lactams results in greater time above the MIC for patients in septic shock [8,15,16,17]. A randomized controlled trial in 2016 comparing continuous infusion dosing of beta lactams with intermittent dosing found higher clinical cure rates in the continuous infusion group [17]. These findings added to a similar 2013 randomized control trial that found increased antibiotic plasma concentrations and improved clinical cure with extended infusion dosing [15]. A 2018 meta-analysis of 22 randomized controlled trials found that prolonged infusion of anti-pseudomonal beta lactams for septic shock resulted in decreased mortality compared to short-term infusion [16]. While limited intravenous access can be a barrier to implementing prolonged infusion strategies, the growing body of evidence to support its use in NSTI patients with septic shock should be considered.

Empiric antibiotic therapy should be narrowed based on culture growth. Linezolid can be discontinued with resolution of shock and lack of MRSA growth typically at 48 h. Broad empiric beta lactams are often exchanged for more narrow ones such as ceftriaxone or ampicillin/sulbactam. Anaerobe coverage is typically continued for the duration of treatment of Type I NSTI, as anaerobes take longer and are more difficult to grow. Several beta lactams include anaerobic coverage, but for those that do not, metronidazole can be added. Yeast may grow in subsequent tissue cultures, selected out with antibiotic exposure, but is rarely considered pathogenic and antifungal coverage is not recommended. No standard antibiotic duration exists, as it is more often guided by confidence in surgical source control and patient clinical improvement. Most of the literature agrees that 7–14 days of antibiotics is adequate for NSTI treatment, often with the end date 24–48 h after last debridement [2,8,11,18]. Recently, our institution noted an increase in Actinomyces growth from Type I NSTI polymicrobial cultures. This correlated with a change in anaerobic identification to matrix-assisted laser desorption/ionization time-of-flight mass spectrometry (MALDI-TOF MS), which had been shown to identify anaerobes with higher accuracy [19]. Classic Actinomycosis is typically a monomicrobial slow contiguous infection in multiple systems including the CNS and pulmonary, abdominal, and musculoskeletal systems and is almost always treated with a prolonged course of therapy. In a retrospective study, it was concluded that standard NSTI antibiotic durations are likely adequate even when actinomyces is identified, as it may not be pathogenic and was possibly underreported with prior anerobic identification techniques [19].

### 2.2. Type II

Type II NSTIs are single-organism-driven and often manifest as the monstrous flesh-eating bacteria of the lay press. The predominant organisms implicated in this type of NSTI are group A streptococcus (*Strep pyogenes*), *Staphylococcus aureus*, Clostridium species and group B, C, and G Streptococci [20]. The notoriety of this type of NSTI is tied to the fact that each of these organisms has the potential to induce something similar to or actual toxic shock syndrome (TSS), which increases both patient morbidity and mortality. As will be discussed, the antimicrobial therapies used should not only be broad enough to target potential pathogens but also to secondarily help limit the bacteria’s production of toxins that contribute to destruction of tissue and the host’s immune response.

This type is often preceded by trauma, surgery, injection drug use, and muscle strain, but occasionally can also present with no history of significant skin injury [8]. The description of the presentation is often a rapidly progressing erythema of the skin, often on an extremity, with accompanying severe pain or, paradoxically, loss of sensation that can occur in response to nerve damage [3,20]. The patient’s clinical presentation is often out of proportion to their skin exam. Common laboratory abnormalities such as hyponatremia, leukocytosis, lactic acidosis, and acute kidney injury are often noted [1,20]. As the disease progresses, the degree of lab abnormalities increases. The skin changes become more pronounced to include crepitus, bullae, and skin necrosis [1]. While the mainstay of treatment involves surgical debridement, antimicrobial therapy also plays a significant role.

Group A Streptococcus (GAS) remains a virulent cause of and represents a significant portion of all Type II NSTI cases [1,8,21]. Data collected on all types of serious GAS infections from 2022 showed that there were approximately 27,000 cases in the United States that year, with NSTIs and TSS representing 5% and 2.3% of these cases, respectively [22,23]. It is worth noting that overall cases of GAS have appeared to be on the rise since 2013, with data from several states showing an increased incidence from 3.6 per 100,000 persons in 2013 to 8.2 per 100,000 persons in 2022 [24]. Preliminary data from CDC also note a rise in cases since 2022 to numbers higher than in the pre-pandemic era [22]. The increase was most notable among older adults and those who experience homelessness and injection drug use [22,24]. There has also been a rise in serious GAS infections in several European nations, which was mostly noted in children below the age of 10 and thought to be related to seasonal rises and co-infection with respiratory viruses [25]. Though GAS TSS and NSTI cases do not appear to be rapidly rising, it is certainly something that will be examined in the next several years with the rise in GAS infections overall.

In an attempt to understand the pathophysiology of GAS, it has been implied that several subtypes may be more closely linked with the development of NSTIs and TSS [26,27]. However, recent research on multiple GAS strains shows an overlap in virulence pathology and suggests the ability to cause invasive disease may not be limited to select strains [27,28]. To date, complement inhibitors, leukocidal toxins, immunoglobulin-binding proteins, and Ig-degrading enzymes have all been identified as facilitating tissue infiltration and avoidance of detection and destruction by the host immune system [8,27]. Finally, and most notably, there is the production of superantigens, which include the Streptococcal pyrogenic exotoxins, the Streptococcal superantigen, and the Streptococcal mitogenic exotoxin Z [27]. These toxins are what induce the overactivation of the host’s immune response, which precipitates the multisystem organ dysfunction syndrome (MODS) and shock seen with TSS [27].

For antimicrobial treatment of confirmed GAS, the antibiotic of choice remains penicillin [2,27]. For treatment of NSTI, the recommendation made by the IDSA is to use both penicillin and clindamycin [2]. Clindamycin monotherapy is not recommended due to rising rates of GAS resistance to both clindamycin and macrolides [24]. However, continuation of clindamycin is still beneficial, not for its antimicrobial use but for its benefit in halting production of superantigens through its ability to inhibit protein synthesis by binding to the 50 S subunit of bacterial ribosomes [2,8]. This helps to slow the process of cytokine storm, shock, and MODS and is effective regardless of organism-specific susceptibility [8]. This was demonstrated by several observational studies, in which there was a reduction in mortality when adding clindamycin [29,30]. There has also been elucidation to suggestion of potential benefit when added to treatment of Type II NSTIs caused by beta hemolytic strep, Clostridial gangrene, and Staphylococcus [8]. In our institution, we start clindamycin therapy early and continue for a two-day duration but will extend in the rare occurrence that end organ markers and vasopressor requirements are showing delayed improvement.

Another inhibitor of protein synthesis, linezolid, has also been shown to cause a similar decrease in toxin production in vitro, as well as in observational data where it showed similar outcomes when used in lieu of clindamycin for GAS NSTIs [31,32,33,34]. Use of linezolid over clindamycin has several potential benefits. For one, in the immediate period of diagnosing NSTIs when broad therapy is used, linezolid may be a good broad Gram-positive drug alternative to vancomycin given that it can provide broad coverage for possible methicillin-resistant Staphylococcus and can provide anti-toxin benefit without needing the addition of clindamycin. It can also be a good alternative option for patients with severe beta lactam allergies and those at risk of AKI where drugs such as vancomycin may want to be avoided. Lastly, clindamycin carries a black-box warning for risk of severe colitis and *Clostridium difficile* infection, making it overall a less favorable choice for treatment or toxin inhibition.

Perhaps the more controversial adjunctive therapy for GAS NSTIs is the use of intravenous immunoglobulin (IVIG), with the presumed benefit being the neutralization of superantigen activity in TSS [3,21,35]. This modality has also been proposed to be beneficial in the treatment of Staphylococcal TSS [3,36]. While many non-randomized studies have demonstrated its benefit in improving outcomes including mortality, there have been no randomized control trials to date that have accomplished the same [29,37,38,39,40]. There are many confounding factors that have likely made it difficult to obtain more definitive answers, such as the rarity of the disease, leading to small sample sizes, the differing doses of IVIG used, the differences in surgical techniques per institution, and the varying use of adjunctive therapies such as HBOT and clindamycin [1,21,29]. A recent meta-analysis, pooling data from randomized and non-randomized studies, was able to demonstrate a significant mortality benefit [41]. It is also worth noting that in vitro data continue to demonstrate a decrease in serum GAS superantigen activity after IVIG administration [35,38]. At our institution, we will administer IVIG in the cases of suspected or confirmed Streptococcal or Staphylococcal TSS in the setting of persistent septic shock and lactemia. Our 3-day dosing protocol is similar to that which was used by Darenberg et al., with 1 g per kg administered on day 1 and then 0.5 g per kg on days 2 and 3 [38].

Staphylococcal NSTI presents in much the same way as Strep infections and contributes to a significant and rising portion of all Type II NSTIs [8]. Like Strep, Staphylococcal TSS stems from superantigen production that leads to cytokine cascade, MODS, and shock [21,36]. The treatment of choice once confirmed is based on whether the organism is methicillin-resistant or -susceptible (Table 1). The use of anti-toxin therapy with clindamycin or linezolid, and, in severe cases, IVIG, is often employed similarly to how it is used for GAS NSTIs.

Finally, Clostridium gas gangrene or myonecrosis is a rare but lethal disease [2,8]. Onset can be either spontaneous or traumatic including in surgical inoculum [2]. Spontaneous cases are typically due to the hematogenous spread of *Clostridium septicum* from the GI tract, often originating in patients that are immunosuppressed or have GI malignancy [2]. Presentation can be severe and similar to Streptococcal or Staphylococcal TSS [20]. Treatment consists of surgery and the antibiotics penicillin plus clindamycin. Dual therapy is used due to rising resistance rates of Clostridium to clindamycin, but also due to the noted increased efficacy of clindamycin therapy in experimental models [2]. However, adjunctive therapy with clindamycin or linezolid and IVIG may also possess potential added benefit for its effects on toxin production and function, but there are limited data on this recommendation [20]. Finally, of all the types of NSTI, there is a sturdy body of literature that suggests hyperbaric oxygen therapy is likely to be of benefit in Clostridium gas gangrene [42].

### 2.3. Type III

Type III NSTIs encompass marine organisms, most notably the two Gram-negative rod species of *Vibrio vulnificus*, typically found in salt water, and *Aeromonas hydrophila*, found in freshwater. Both demonstrate seasonal variations, with increased cases in warmer months. Rising sea surface temperatures related to climate change have been shown to alter bacterial saturation, biofilm production, and resistance mechanisms [43,44,45]. Climate change has also led to an increase in disease acquisition within parts of the world where disease had been less commonly seen [43,44]. Management of these types of infections is like that of other types of NSTI in that early and aggressive surgical intervention is necessary. Antimicrobial therapy, as in the case of Type I and II disease, is optimized to the best-known and most direct regimens once confirmed with culture data.

*Vibrio vulnificus* NSTI remains a highly lethal infection [43,46]. It is typically introduced via the GI tract with ingestion of raw seafood or via a break in the skin or wound with exposure to salt or brackish water, or to seafood [44]. While individuals with liver disease, diabetes, immunosuppression, and alcohol use disorder seem to be at higher risk of contracting these infections, the disease is not exclusive to these subgroups. One of the proposed theoretical predispositions of infection in patients with alcohol use and liver disease may be due to high transferrin iron blood concentrations in this population, to which the organism can bind to and use to promote growth [46]. *V. vulnificus* possesses several factors that contribute to its pathogenicity through enhanced adhesion to mammalian cells as well as to the production of toxins that can induce MODS and shock similar to what is seen in TSS [44]. Antimicrobial treatment consists of either a dual-therapy regimen with ceftriaxone and doxycycline or monotherapy with a fluroquinolone. This was due to prior studies showing decreased mortality for severe infections when combining a third-generation cephalosporin and a tetracycline, as well as a reduction in mortality with a fluoroquinolone alone [46]. While early antimicrobial therapy is usually broad, identifying if the patient has a history of water exposure is important to determine if tetracycline, fluroquinolone, and/or cephalosporin therapy should be included in the empiric regimen to cover for the potential of Vibrio species.

Aeromonas, like Vibrio, is a Gram-negative species found in various aquatic environments, soil, sewage, and some foods including produce and fish [4,47]. Clinical manifestations range from GI illnesses to NSTIs, with NSTIs developing mainly in patients with underlying risk factors such as immunosuppression, diabetes, renal disease, and liver cirrhosis [4]. Mortality rates of NSTI can be as high as 75–100% when seen with bacteremia and MODS [4]. Antimicrobial resistance varies by strain and whether isolates are environmental or clinical. Generally, resistance is chromosomally mediated, but similar to other Gram-negative bacteria, Aeromonas is capable of beta lactamase enzyme production that disables common beta lactam antibiotics. Reasonable empiric regimens that can be employed while awaiting susceptibility results include combination therapy with cefepime and fluoroquinolone or doxycycline, as our service has anecdotally noted higher piperacillin/tazobactam resistance [47]. As above, identifying an epidemiologic link to water is critical in the decision-making pathway.

### 2.4. Type IV

Type IV NSTIs encompass various fungal etiologies (Table 1) that are mainly introduced via trauma [3]. Host immune function plays a role in the organism, severity, and survivability of the infection [2,3]. Treatment is beyond the scope of this article but often involves referral to a specialty surgical center and expertise in optimizing antifungal therapy.

## 3. Conclusions

Overall, NSTIs remain an emergent medical condition due to their aggressive nature. Clinical suspicion should be high in any NSTI patient, as there is variable clinical presentation and high morbidity and mortality. The two-pronged approach of early antimicrobial treatment and early surgical intervention is required for cure, as antimicrobials do not penetrate dead and dying tissue well. The samples obtained and sent for culture are also profoundly helpful in determining the specific type of NSTI. This microbiological data are ultimately used to optimize the antimicrobial treatment and cure the infection. Our review hopes to bring to attention some of the newer treatment trends for NSTIs, some of which include the use of linezolid over clindamycin for toxin suppression, optimizing antibiotic concentrations via extended infusion dosing, and adjusting antimicrobial selection and dosing to mitigate AKI risk in patients with obesity. Lastly, when it comes to antibiotic duration, it is often acceptable to finish antibiotic therapy 24–48 h following the final debridement. Other approaches for NSTI treatment are under development. There are ongoing efforts to create a GAS vaccine, which would hopefully reduce the occurrence and the severity of GAS infections, including NSTI [28]. Additionally, plasmapheresis may be an emerging treatment of interest, as it has already been used for other infections [48]. As of now, the standard of care remains the two-pronged approach of targeted antimicrobials and surgical intervention.

## Figures and Tables

**Table 1:** Pathogens and Standard Antibiotic Regimens for Necrotizing Soft Tissue Infections.

Type	Most Common Pathogens	Antimicrobial Therapies
I	Most likely aerobes: *Staphylococcal aureus*, *Escherichia coli*, Klebsiella, Proteus, Enterococcus, Enterobacter, alpha hemolytic strep spp. Most likely anaerobes: Bacteroides, Prevotella, Peptostreptococcus, Clostridium, Actinomyces Less likely aerobes, dependent on environment and antibiotic exposure: Pseudomonas, Serratia, Morganella, Acinetobacter	Empiric: Linezolid plus Piperacillin/Tazobactam Definitive Treatment: Tailor based on culture growth and antibiotic susceptibility testing
II	Beta hemolytic strep Methicillin-resistant *Staphylococcal aureus* (MRSA) Methicillin-susceptible *Staphylococcal aureus* (MSSA) Clostridium sp.	Penicillin G * Vancomycin, Daptomycin, Linezolid * Cefazolin or Oxacillin * Penicillin G *
III	Vibrio, Aeromonas	Empiric: Cefepime plus doxycycline or fluoroquinolone Vibrio: Ceftriaxone plus doxycycline or quinolone alone Aeromonas: Narrow based on antimicrobial susceptibility testing
IV	Candida species in immunocompromised patients Mucoraceous molds/Aspergillus in immunocompetent patients/trauma	Empiric: Micafungin Empiric: Amphotericin B

* If septic shock is present, this should be accompanied with anti-toxin therapy using either clindamycin or linezolid.
